# 2,3-Dibromo-6-meth­oxy-4-[(phenethyl­amino)­methyl­idene]cyclo­hexa-2,5-dien-1-one methanol monosolvate

**DOI:** 10.1107/S1600536811052895

**Published:** 2011-12-14

**Authors:** Rong-Bao Ge, Yue-Hu Chen, Feng-Ting Wang, Shuang-Shuang Wang, Shao-Song Qian

**Affiliations:** a School of Life Sciences, ShanDong University of Technology, ZiBo 255049, People’s Republic of China

## Abstract

In the title compound, C_16_H_15_Br_2_NO_2_·CH_4_O, the mean planes of the substituted cyclo­hexa-2,5-dien-1-one and phenyl rings are almost parallel [dihedral angle = 7.84 (4)°]. The crystal packing is stabilized by N—H⋯O hydrogen bonds generating infinite [101] chains. The methanol solvent mol­ecules are connected with the main species by O—H⋯O inter­actions.

## Related literature

For background to bromo­phenols and their bioactivity, see: Liu *et al.* (2011 ▶). For related structures, see: Palmer *et al.* (1973 ▶); Li *et al.* (1995 ▶); Huang *et al.* (2006 ▶). For structural and theoretical aspects on the keto-enol equilibrium of salicyl­aldehyde Schiff bases, see: Chatziefthimiou *et al.* (2006 ▶).
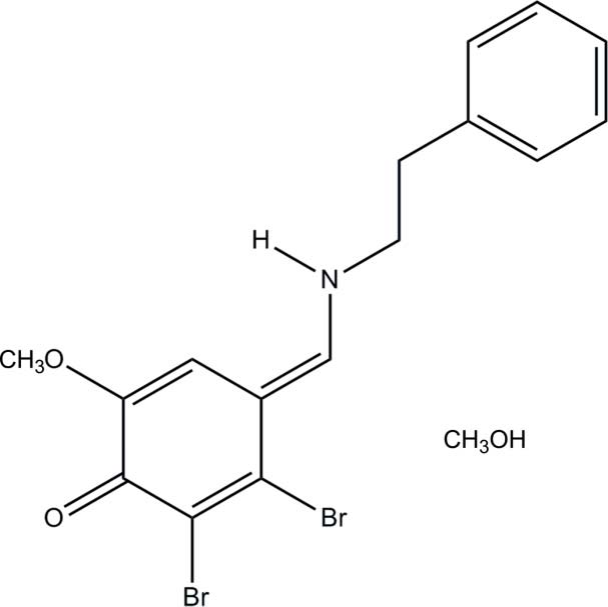


## Experimental

### 

#### Crystal data

C_16_H_15_Br_2_NO_2_·CH_4_O
*M* *_r_* = 445.15
Monoclinic, 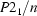

*a* = 8.752 (6) Å
*b* = 16.308 (10) Å
*c* = 13.001 (8) Å
β = 104.047 (6)°
*V* = 1800 (2) Å^3^
*Z* = 4
Mo *K*α radiation
μ = 4.52 mm^−1^
*T* = 296 K
0.25 × 0.22 × 0.20 mm

#### Data collection

Bruker APEXII CCD diffractometer
Absorption correction: multi-scan (*SADABS*; Bruker, 2004 ▶) *T*_min_ = 0.337, *T*_max_ = 0.406
8282 measured reflections
3292 independent reflections
1674 reflections with *I* > 2σ(*I*)
*R*_int_ = 0.072

#### Refinement

*R*[*F* ^2^ > 2σ(*F* ^2^)] = 0.051
*wR*(*F* ^2^) = 0.133
*S* = 1.00
3292 reflections
211 parameters
H-atom parameters constrained
Δρ_max_ = 0.62 e Å^−3^
Δρ_min_ = −0.60 e Å^−3^

### 

Data collection: *APEX2* (Bruker, 2004 ▶); cell refinement: *SAINT* (Bruker, 2004 ▶); data reduction: *SAINT*; program(s) used to solve structure: *SHELXS97* (Sheldrick, 2008 ▶); program(s) used to refine structure: *SHELXL97* (Sheldrick, 2008 ▶); molecular graphics: *SHELXTL* (Sheldrick, 2008 ▶); software used to prepare material for publication: *SHELXTL*.

## Supplementary Material

Crystal structure: contains datablock(s) global, I. DOI: 10.1107/S1600536811052895/zq2143sup1.cif

Structure factors: contains datablock(s) I. DOI: 10.1107/S1600536811052895/zq2143Isup2.hkl

Supplementary material file. DOI: 10.1107/S1600536811052895/zq2143Isup3.cml

Additional supplementary materials:  crystallographic information; 3D view; checkCIF report

## Figures and Tables

**Table 1:** Hydrogen-bond geometry (Å, °)

*D*—H⋯*A*	*D*—H	H⋯*A*	*D*⋯*A*	*D*—H⋯*A*
N1—H1⋯O1^i^	0.86	1.93	2.731 (7)	154
O3—H3⋯O1^ii^	0.82	2.05	2.786 (8)	150

Symmetry codes: (i) 
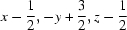
; (ii) 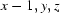.

**Fig. 1.**
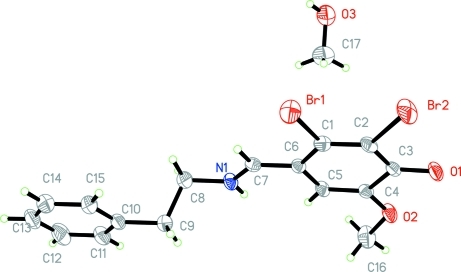
Molecular structure of the title compound. Displacement ellipsoids are drawn at the 30% probability level.

**Fig. 2.**
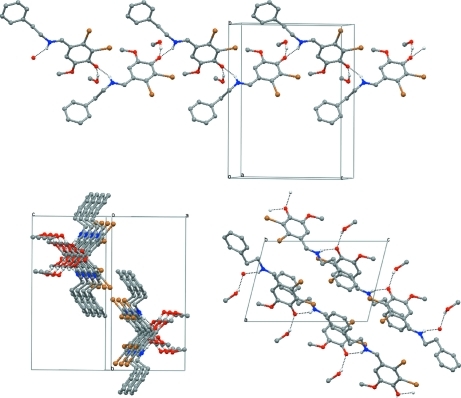
Different views of the crystal packing showing N–H···O and O–H···O hydrogen bonds (dashed lines).

## supplementary crystallographic information

### Comment

The 2,3-dibromo-4-hydroxy-5-methoxybenzaldehyde acts as an important precursor for the synthesis of bromophenols, which have been reported to possess a variety of biological activities (Liu *et al.*, 2011). As an extension of our work on the 2,3-dibromo-4-hydroxy-5-methoxybenzaldehyde, the title compound was synthesized by condensing 2,3-dibromo-4-hydroxy-5-methoxybenzaldehyde with phenethylamine, and attempts to investigate its biological activities were carried out.

In the crystal structure of the title compound, C_16_H_15_Br_2_NO_2_.CH_4_O, the mean planes of the substituted cyclohexa-2,5-dien-1-one and phenyl rings are almost parallel [dihedral angle = 7.84 (4)°]. Difference Fourier maps clearly showed that the N1 atom is protonated rather than the O1 atom indicating a methylidenecyclohexa-2,5-dien-1-one skeleton (Palmer *et al.*, 1973; Huang *et al.*, 2006; Chatziefthimiou *et al.*, 2006). The N1 and O1 atoms are connected *via* a short intermolecular N—H···O hydrogen bond [N1···O1^i^ = 2.731 (7) Å; (i) = *x* - 1/2, -*y* + 3/2, *z* - 1/2] generating infinite one-dimensional [101] chains (Chatziefthimiou *et al.*, 2006). The solvent molecules of methanol are connected with the main species by O3—H3···O1^ii^ interactions [O3···O1^ii^ = 2.786 (8) Å; (ii) = *x* - 1, *y*, *z*].

### Experimental

2,3-Dibromo-4-hydroxy-5-methoxybenzaldehyde (0.31 g) and phenethylamine (0.12 g) were dissolved in methanol (20 ml). The mixture was stirred at room temperature for 30 min to give a clear solution. Keeping the solution in air for 5 days, yellow block-shaped single crystals suitable for X-ray diffraction analysis were obtained at the bottom of the vessel.

### Refinement

All H atoms were placed in geometrical positions and constrained to ride on their parent atoms, with C—H = 0.93 Å and *U*_iso_(H) = 1.2*U*_eq_(C) for aromatic H atoms, with C—H = 0.97 Å and *U*_iso_(H) = 1.2*U*_eq_(C) for methylene H atoms, with C—H = 0.96 Å and *U*_iso_(H) = 1.5*U*_eq_(C) for methyl H atoms, and with N—H = 0.86 Å and *U*_iso_(H) = 1.2*U*_eq_(C) for the amino H atom.

### Crystal data

**Table:**

C_16_H_15_Br_2_NO_2_·CH_4_O	*Z* = 4
*M**_r_* = 445.15	*F*(000) = 888
Monoclinic, *P*2_1_/*n*	*D*_x_ = 1.643 Mg m^−^^3^
Hall symbol: -P 2yn	Mo *K*α radiation, λ = 0.71073 Å
*a* = 8.752 (6) Å	µ = 4.52 mm^−^^1^
*b* = 16.308 (10) Å	*T* = 296 K
*c* = 13.001 (8) Å	Block, yellow
β = 104.047 (6)°	0.25 × 0.22 × 0.20 mm
*V* = 1800 (2) Å^3^	

### Data collection

**Table:**

Bruker APEXII CCD diffractometer	3292 independent reflections
Radiation source: fine-focus sealed tube	1674 reflections with *I* > 2σ(*I*)
graphite	*R*_int_ = 0.072
φ and ω scans	θ_max_ = 25.5°, θ_min_ = 2.5°
Absorption correction: multi-scan (*SADABS*; Bruker, 2004)	*h* = −10→10
*T*_min_ = 0.337, *T*_max_ = 0.406	*k* = −19→14
8282 measured reflections	*l* = −12→15

### Refinement

**Table:**

Refinement on *F*^2^	Secondary atom site location: difference Fourier map
Least-squares matrix: full	Hydrogen site location: inferred from neighbouring sites
*R*[*F*^2^ > 2σ(*F*^2^)] = 0.051	H-atom parameters constrained
*wR*(*F*^2^) = 0.133	*w* = 1/[σ^2^(*F*_o_^2^) + (0.0388*P*)^2^ + 2.4528*P*] where *P* = (*F*_o_^2^ + 2*F*_c_^2^)/3
*S* = 1.00	(Δ/σ)_max_ = 0.001
3292 reflections	Δρ_max_ = 0.62 e Å^−^^3^
211 parameters	Δρ_min_ = −0.60 e Å^−^^3^
0 restraints	Extinction correction: *SHELXL97* (Sheldrick, 2008)
Primary atom site location: structure-invariant direct methods	Extinction coefficient: 0.0103 (10)

### Special details

**Table:**

Geometry. All e.s.d.'s (except the e.s.d. in the dihedral angle between two l.s. planes) are estimated using the full covariance matrix. The cell e.s.d.'s are taken into account individually in the estimation of e.s.d.'s in distances, angles and torsion angles; correlations between e.s.d.'s in cell parameters are only used when they are defined by crystal symmetry. An approximate (isotropic) treatment of cell e.s.d.'s is used for estimating e.s.d.'s involving l.s. planes.
Refinement. Refinement of *F*^2^ against ALL reflections. The weighted *R*-factor *wR* and goodness of fit *S* are based on *F*^2^, conventional *R*-factors *R* are based on *F*, with *F* set to zero for negative *F*^2^. The threshold expression of *F*^2^ > σ(*F*^2^) is used only for calculating *R*-factors(gt) *etc*. and is not relevant to the choice of reflections for refinement. *R*-factors based on *F*^2^ are statistically about twice as large as those based on *F*, and *R*- factors based on ALL data will be even larger.

### Fractional atomic coordinates and isotropic or equivalent isotropic displacement parameters (Å^2^)

**Table:**

	*x*	*y*	*z*	*U*_iso_*/*U*_eq_	
Br1	0.50517 (10)	0.54245 (5)	0.37790 (6)	0.0638 (3)	
Br2	0.78645 (10)	0.65471 (6)	0.52622 (6)	0.0728 (3)	
O1	0.9067 (5)	0.7788 (3)	0.3957 (3)	0.0515 (13)	
O2	0.8183 (6)	0.8146 (3)	0.1919 (3)	0.0544 (13)	
O3	0.1533 (9)	0.8797 (5)	0.3731 (6)	0.129 (3)	
H3	0.1090	0.8384	0.3873	0.193*	
N1	0.3484 (6)	0.6175 (3)	0.0466 (4)	0.0429 (15)	
H1	0.3848	0.6572	0.0162	0.051*	
C1	0.5918 (7)	0.6306 (4)	0.3183 (5)	0.0401 (17)	
C2	0.7125 (8)	0.6750 (4)	0.3797 (5)	0.0404 (17)	
C3	0.7919 (8)	0.7392 (4)	0.3400 (5)	0.0373 (16)	
C4	0.7342 (7)	0.7544 (4)	0.2277 (5)	0.0363 (16)	
C5	0.6117 (7)	0.7126 (4)	0.1670 (5)	0.0385 (17)	
H5	0.5764	0.7260	0.0956	0.046*	
C6	0.5359 (7)	0.6489 (4)	0.2093 (5)	0.0347 (16)	
C7	0.4111 (7)	0.6048 (4)	0.1455 (5)	0.0375 (16)	
H7	0.3694	0.5623	0.1778	0.045*	
C8	0.2192 (8)	0.5689 (5)	−0.0177 (5)	0.054 (2)	
H8A	0.1369	0.6054	−0.0553	0.065*	
H8B	0.1749	0.5342	0.0283	0.065*	
C9	0.2756 (8)	0.5160 (5)	−0.0969 (6)	0.061 (2)	
H9A	0.3238	0.5506	−0.1410	0.074*	
H9B	0.3551	0.4781	−0.0591	0.074*	
C10	0.1429 (8)	0.4687 (5)	−0.1657 (6)	0.0500 (19)	
C11	0.0749 (9)	0.4913 (5)	−0.2688 (6)	0.061 (2)	
H11	0.1126	0.5374	−0.2968	0.074*	
C12	−0.0470 (10)	0.4476 (6)	−0.3314 (7)	0.080 (3)	
H12	−0.0906	0.4644	−0.4007	0.096*	
C13	−0.1036 (10)	0.3802 (6)	−0.2923 (7)	0.070 (3)	
H13	−0.1845	0.3497	−0.3348	0.084*	
C14	−0.0414 (9)	0.3580 (5)	−0.1913 (7)	0.067 (2)	
H14	−0.0813	0.3124	−0.1636	0.080*	
C15	0.0808 (8)	0.4018 (5)	−0.1279 (6)	0.055 (2)	
H15	0.1215	0.3854	−0.0581	0.066*	
C17	0.2388 (12)	0.8606 (7)	0.3029 (8)	0.108 (4)	
H17A	0.3099	0.9047	0.2992	0.162*	
H17B	0.1695	0.8520	0.2342	0.162*	
H17C	0.2979	0.8115	0.3254	0.162*	
C16	0.7803 (10)	0.8284 (5)	0.0801 (6)	0.075 (3)	
H16A	0.7963	0.7788	0.0444	0.112*	
H16B	0.8468	0.8708	0.0639	0.112*	
H16C	0.6721	0.8449	0.0568	0.112*	

### Atomic displacement parameters (Å^2^)

**Table:**

	*U*^11^	*U*^22^	*U*^33^	*U*^12^	*U*^13^	*U*^23^
Br1	0.0743 (6)	0.0668 (6)	0.0470 (5)	−0.0142 (5)	0.0082 (4)	0.0219 (4)
Br2	0.0894 (7)	0.0934 (7)	0.0260 (4)	−0.0155 (5)	−0.0043 (4)	0.0126 (4)
O1	0.059 (3)	0.059 (3)	0.031 (3)	−0.010 (3)	0.001 (2)	−0.010 (2)
O2	0.072 (3)	0.058 (3)	0.031 (3)	−0.023 (3)	0.008 (3)	−0.003 (2)
O3	0.127 (6)	0.181 (8)	0.090 (5)	−0.085 (6)	0.049 (5)	−0.052 (6)
N1	0.047 (3)	0.048 (4)	0.029 (3)	−0.004 (3)	0.001 (3)	−0.001 (3)
C1	0.039 (4)	0.047 (4)	0.033 (4)	0.008 (3)	0.008 (3)	0.006 (3)
C2	0.051 (4)	0.046 (4)	0.023 (3)	0.007 (4)	0.006 (3)	0.003 (3)
C3	0.042 (4)	0.041 (4)	0.028 (4)	0.003 (3)	0.007 (3)	−0.006 (3)
C4	0.043 (4)	0.038 (4)	0.029 (4)	−0.001 (3)	0.012 (3)	0.001 (3)
C5	0.041 (4)	0.048 (4)	0.022 (3)	0.003 (3)	−0.001 (3)	0.001 (3)
C6	0.036 (4)	0.038 (4)	0.029 (4)	0.002 (3)	0.006 (3)	0.000 (3)
C7	0.043 (4)	0.040 (4)	0.031 (4)	0.002 (3)	0.011 (3)	0.002 (3)
C8	0.051 (4)	0.067 (5)	0.042 (4)	−0.008 (4)	0.009 (4)	−0.009 (4)
C9	0.047 (5)	0.081 (6)	0.057 (5)	−0.012 (4)	0.014 (4)	−0.024 (4)
C10	0.040 (4)	0.057 (5)	0.051 (5)	0.001 (4)	0.007 (4)	−0.020 (4)
C11	0.076 (6)	0.060 (5)	0.046 (5)	−0.005 (5)	0.012 (5)	−0.008 (4)
C12	0.085 (7)	0.089 (7)	0.052 (5)	0.006 (6)	−0.008 (5)	−0.019 (5)
C13	0.060 (6)	0.070 (6)	0.073 (7)	0.001 (5)	0.004 (5)	−0.033 (5)
C14	0.062 (5)	0.062 (6)	0.080 (7)	−0.007 (4)	0.022 (5)	−0.018 (5)
C15	0.055 (5)	0.060 (5)	0.048 (5)	−0.006 (4)	0.010 (4)	−0.009 (4)
C17	0.111 (9)	0.129 (9)	0.095 (8)	−0.031 (7)	0.048 (7)	−0.020 (7)
C16	0.096 (7)	0.081 (6)	0.043 (5)	−0.031 (5)	0.010 (5)	0.012 (4)

### Geometric parameters (Å, °)

**Table:**

Br1—C1	1.878 (7)	C8—H8B	0.9700
Br2—C2	1.886 (6)	C9—C10	1.497 (9)
O1—C3	1.263 (7)	C9—H9A	0.9700
O2—C4	1.374 (7)	C9—H9B	0.9700
O2—C16	1.428 (8)	C10—C15	1.363 (10)
O3—C17	1.350 (10)	C10—C11	1.377 (9)
O3—H3	0.8200	C11—C12	1.373 (10)
N1—C7	1.286 (7)	C11—H11	0.9300
N1—C8	1.465 (8)	C12—C13	1.355 (11)
N1—H1	0.8600	C12—H12	0.9300
C1—C2	1.366 (9)	C13—C14	1.344 (11)
C1—C6	1.414 (8)	C13—H13	0.9300
C2—C3	1.422 (9)	C14—C15	1.380 (10)
C3—C4	1.445 (8)	C14—H14	0.9300
C4—C5	1.352 (8)	C15—H15	0.9300
C5—C6	1.414 (8)	C17—H17A	0.9600
C5—H5	0.9300	C17—H17B	0.9600
C6—C7	1.399 (8)	C17—H17C	0.9600
C7—H7	0.9300	C16—H16A	0.9600
C8—C9	1.514 (9)	C16—H16B	0.9600
C8—H8A	0.9700	C16—H16C	0.9600
			
C4—O2—C16	116.5 (5)	C8—C9—H9A	109.3
C17—O3—H3	109.5	C10—C9—H9B	109.3
C7—N1—C8	124.5 (6)	C8—C9—H9B	109.3
C7—N1—H1	117.7	H9A—C9—H9B	108.0
C8—N1—H1	117.7	C15—C10—C11	116.6 (7)
C2—C1—C6	120.3 (6)	C15—C10—C9	121.1 (7)
C2—C1—Br1	119.8 (5)	C11—C10—C9	122.3 (7)
C6—C1—Br1	119.9 (5)	C12—C11—C10	121.9 (8)
C1—C2—C3	123.7 (6)	C12—C11—H11	119.0
C1—C2—Br2	121.6 (5)	C10—C11—H11	119.0
C3—C2—Br2	114.7 (5)	C13—C12—C11	120.0 (8)
O1—C3—C2	124.0 (6)	C13—C12—H12	120.0
O1—C3—C4	121.7 (6)	C11—C12—H12	120.0
C2—C3—C4	114.3 (6)	C14—C13—C12	119.1 (8)
C5—C4—O2	125.2 (6)	C14—C13—H13	120.4
C5—C4—C3	122.5 (6)	C12—C13—H13	120.4
O2—C4—C3	112.3 (5)	C13—C14—C15	121.0 (8)
C4—C5—C6	121.6 (6)	C13—C14—H14	119.5
C4—C5—H5	119.2	C15—C14—H14	119.5
C6—C5—H5	119.2	C10—C15—C14	121.2 (7)
C7—C6—C5	121.3 (6)	C10—C15—H15	119.4
C7—C6—C1	121.1 (6)	C14—C15—H15	119.4
C5—C6—C1	117.6 (6)	O3—C17—H17A	109.5
N1—C7—C6	126.3 (6)	O3—C17—H17B	109.5
N1—C7—H7	116.9	H17A—C17—H17B	109.5
C6—C7—H7	116.9	O3—C17—H17C	109.5
N1—C8—C9	111.2 (6)	H17A—C17—H17C	109.5
N1—C8—H8A	109.4	H17B—C17—H17C	109.5
C9—C8—H8A	109.4	O2—C16—H16A	109.5
N1—C8—H8B	109.4	O2—C16—H16B	109.5
C9—C8—H8B	109.4	H16A—C16—H16B	109.5
H8A—C8—H8B	108.0	O2—C16—H16C	109.5
C10—C9—C8	111.5 (6)	H16A—C16—H16C	109.5
C10—C9—H9A	109.3	H16B—C16—H16C	109.5

### Hydrogen-bond geometry (Å, °)

**Table:**

*D*—H···*A*	*D*—H	H···*A*	*D*···*A*	*D*—H···*A*
N1—H1···O1^i^	0.86	1.93	2.731 (7)	154.
O3—H3···O1^ii^	0.82	2.05	2.786 (8)	150.

Symmetry codes: (i) *x*−1/2, −*y*+3/2, *z*−1/2; (ii) *x*−1, *y*, *z*.
